# Translation and Validation of the City Birth Trauma Scale With Lithuanian Postpartum Women: Findings and Initial Results

**DOI:** 10.1177/01632787241239339

**Published:** 2024-03-12

**Authors:** Olga Riklikienė, Gabija Jarašiūnaitė-Fedosejeva, Ernesta Sakalauskienė, Žydrūnė Luneckaitė, Susan Ayers

**Affiliations:** 1 230647 Lithuanian University of Health Sciences, Lithuania; 2 105468 Vytautas Magnus University, Lithuania; 3 City University of London, UK

**Keywords:** perinatal mental health, trauma, City BiTS, validation, Lithuania

## Abstract

The childbirth experience and birth-related trauma are influenced by various factors, including country, healthcare system, a woman’s history of traumatic experiences, and the study’s design and instruments. This study aimed to validate the City Birth Trauma scale for Lithuanian women post-childbirth. Using a descriptive, cross-sectional survey with a nonprobability sample of 794 women who gave birth from 2020–2021, the study found good validity, reliability, and presented the prevalence of birth-related stress symptoms. A bifactor model, consisting of a general birth trauma factor and two specific factors for birth-related symptoms and general symptoms of PTSD, showed the best model fit. The Lithuanian version of the City Birth Trauma scale can be effectively used in research and clinical practice to identify birth-related trauma symptoms in women after giving birth.

## Introduction

Pregnancy and childbirth bring about significant physiological, psychological, social, and spiritual changes for women. It’s a period marked by tremendous transformation, transition, and adjustment to a new phase of life for women (Olza et al., 2018). While childbirth is a positive experience for some women, studies indicate that 20–48% of women find giving birth psychologically traumatic (Ayers et al., 2009; Dimassi et al., 2020; Nagle et al., 2022). Meta-analyses of research have revealed that 3–4% of women who have given birth develop posttraumatic stress disorder (PTSD) (Grekin & O’Hara, 2014; Yildiz et al., 2017), and clinically significant symptoms of PTSD are present in up to 16.8% of women. A positive childbirth experience promotes women’s health, both during and beyond the perinatal period (Leinweber et al., 2022).

Traumatic birth experiences can be defined in various ways. In 2022, a woman-centered and inclusive definition of traumatic childbirth experiences was provided as ‘a woman’s experience of interactions and/or events directly related to childbirth that caused overwhelming distressing emotions and reactions, leading to short- and/or long-term negative impacts on a woman’s health and well-being’ (Leinweber et al., 2023). Traumatic childbirth is a complex concept that may be related to the mode of birth and the behavior and care of health professionals (Ayers, 2014). During the perinatal period, women expect women-centered care with support and close attention from healthcare staff (Tanaka et al., 2020). However, this is not always provided, with some women reporting physical abuse and disrespectful behavior in the form of scolding, insults, discrimination, or unconsented care during labor (Dorairajan et al., 2020). When birth experiences fall short of expectations, they are associated with lower birth satisfaction and a potentially increased risk of developing postnatal PTSD (Webb et al., 2021). Therefore, the quality of care and women-centered, trauma-informed care policies are essential for women to feel supported, in control, safe, and respected during labor and childbirth (Coo et al., 2023; Leinweber et al., 2022; Mayopoulos et al., 2021).

Little research on the topic of birth-related trauma has been conducted in Lithuania, and there are no validated measures of birth-related PTSD and stress symptoms for use in the country. In this study, our goal was to translate, adapt, psychometrically test, and validate the City Birth Trauma Scale (City BiTS) in the Lithuanian language and culture. A secondary aim was to report on the prevalence of birth-related stress symptoms among the study population.

The selection of this measure for validation was based on several reasons. First, the European nature of the instrument, developed and validated in 2018 in the United Kingdom (Ayers et al., 2018), made it well-suited for adaptation in other European countries. Second, this scale aligns with DSM-5 criteria for PTSD and is specific to childbirth, rather than providing a general assessment of traumatic experiences. Third, the instrument is available in multiple languages and maintains a stable factorial structure, facilitating the exploration of the phenomenon within a particular cultural context while allowing for international comparisons. For instance, the City BiTS has been successfully translated and validated in languages such as Turkish (Bayrı Bingöl et al., 2021), Brazilian Portuguese (Osório et al., 2021), Hebrew (Handelzalts et al., 2018), French (Sandoz et al., 2021), Croatian (Nakić Radoš et al., 2019), Spanish (Caparros-Gonzalez et al., 2021), and German (Weigl et al., 2021). The widespread use of this tool beyond its Anglo-Saxon country of origin indicates its potential for successful validation in other languages. Finally, an advantage of the City BiTS scale is that most of its items consist of short, clear statements describing symptoms, which is crucial for the instrument’s practicality and comprehensibility.

## Methods

### Study Design

For a descriptive, cross-sectional survey the participants were recruited through invitation leaflets and social media. Invitation leaflets were distributed in person to women who had given birth at two institutions: the Hospital of Lithuanian Health Sciences Kauno Clinics and the Maternity home named after P. Mažylis. Additionally, invitations to participate were posted on Facebook groups for mothers who had recently given birth, such as ‘Mothers of 2020/2021’ and ‘Babies of July 2021,’ among others. Invitations were also shared on the Facebook pages of several maternity wards in university, regional, and district healthcare institutions.

### Study Population

A convenience non-probability sample of 753 women who gave birth between 2020 and 2021 participated in the study. The inclusion criteria were women aged 18 or older, proficient in the Lithuanian language, and having given birth 1–12 months ago, who voluntarily agreed to participate in a survey. Exclusion criteria included women younger than 18 years old, those who had given birth less than 1 month or more than 12 months ago, those who experienced stillbirth or newborn death during the study period, those who did not understand the Lithuanian language, and those who refused to participate in the survey.

### Measures

The City Birth Trauma Scale was developed by Ayers, Wright & Thornton in 2018. It is designed based on the diagnostic criteria for PTSD outlined in the DSM-5 and is utilized to assess the presence of PTSD following childbirth. The initial two questions are designed to determine whether the event meets the criteria for a traumatic stressor, as outlined in diagnostic criterion A. These questions inquire about concerns regarding the serious harm or death of the woman or the baby, and responses are scored on a yes/no scale.

To evaluate symptoms, the scale employs 22 items categorized into four groups: re-experiencing, avoidance, negative cognitions and mood, and hyperarousal. Participants are asked to report the frequency of these symptoms experienced over the past week, with scores ranging from zero (‘not at all’) to 3 (‘5 or more times’), where higher scores indicate greater severity of PTSD symptoms (Ayers et al., 2018). Additionally, two items assess when these symptoms began and their duration. Towards the end of the assessment, three items inquire about distress, disability, and potential physical causes. Responses to distress and disability questions are rated as yes/no/sometimes, while responses to the question regarding physical causes are rated as yes/no/maybe.

The original City Birth Trauma Scale demonstrated high internal consistency (Cronbach’s α = 0.92) and exhibited a two-factor structure that collectively explained 56% of the variability. Specifically, symptoms related to childbirth contributed to 40.8% of the variance, while general symptoms accounted for 15.5% of the variance (Ayers et al., 2018).

### Translation Procedure

The translation of the City BiTS adhered to established principles for instrument translation and adaptation (Maneesriwongul & Dixon, 2004). Two native Lithuanian translators, one a professional translator and the other a nursing researcher, independently translated the scale from English to Lithuanian. A nurse educator then compared both translated Lithuanian versions, and any discrepancies were resolved through discussions with the initial translator to reach a consensus. To address linguistic and cultural differences in translation, experts with relevant backgrounds in midwifery, maternity care, psychology, psychiatry, and proficiency in the Lithuanian language were consulted. The consolidated Lithuanian version of the scale was agreed upon after these consultations.

Back translation of the City BiTS from Lithuanian to English was carried out by English language specialists. Equivalency and congruence were ensured by comparing the original and back-translated English versions of the instrument. This process involved collaboration with the original instrument's author, Ayers, to resolve any discrepancies or differences. Additionally, a language specialist reviewed the Lithuanian language style, syntax, and grammar multiple times during the translation procedure, both after the initial translation from English to Lithuanian and after the equivalency checking.

### Ethical Considerations

The Regional Committee on Biomedical Research Ethics granted permission to conduct the study (No. BE-2-73). Prior to completing the questionnaire, participants were presented with an informed consent form containing information about the study’s objectives, the research team, inclusion criteria for study participants, potential risks, benefits of participation, participant rights, as well as details regarding anonymity and confidentiality. Participants were then asked to indicate their consent to participate by checking a designated box.

### Data Analysis

To validate City BiTS-LT, face validity, content validity, construct validity, and reliability were assessed. Construct validity was evaluated through exploratory factor analysis (EFA) and confirmatory factor analysis (CFA). EFA used Principal Axis Factoring, with matrix factorization confirmed via Bartlett’s Test of Sphericity and Kaiser-Meyer-Olkin (KMO) Test. EFA extraction relied on eigenvalues > 1, with Promax rotation. CFA, treating ordinal variables as categorical, employed the WLSMV estimator (Kelloway, 2015; Li, 2015). Fit indices, including Chi-squared, SRMR, RMSEA, CFI, and TLI, assessed model fit. Discriminant validity was tested with Mann-Whitney U and Kruskal-Wallis tests. Reliability was assessed with Cronbach’s alpha and McDonald’s omega. EFA, discriminant validity, and reliability analysis using Cronbach’s alpha were conducted in IBM SPSS Statistics 23.0. McDonald’s omega was calculated using the OMEGA macro by A. F. Hayes. CFA was performed using Mplus 8.6 software. Cronbach’s alpha > 0.6 indicated acceptable internal consistency (Bland & Altman, 1997).

## Results

### Study Sample Characteristics

The participants’ ages ranged from 18 to 44 years, with a mean age of 30.18 years (SD = 4.86). On average, the mothers had given birth five months ago (SD = 3.19, range: 1–12 months). Most of the women were first-time mothers (60.8%), categorized as primiparous, while 30.1% had given birth for the second time, and 8.9% had given birth for the third time or more. The average gestation period at the time of birth was 38.63 weeks (SD = 3.12, range: 22–42 weeks). Of these, 88.2% had term deliveries (after 37 weeks), while 11.8% experienced preterm births.

The majority of women gave birth through spontaneous vaginal delivery (73.6%). Among these, 42.0% did not receive pain relief during labor and birth, and approximately one-third of women (31.6%) opted for epidural anesthesia. A small percentage (6.5%) had planned caesarean sections, 3.0% had instrumental vaginal deliveries, and 17.0% underwent emergency caesarean sections. Of the sample, 10.0% reported that their childbirth experience was traumatic. As for the newborns, the majority (94%) were reported as healthy, while 6% of the participants stated that their newborns had health issues. In terms of the respondents’ own health, the majority reported very good health (30.1%) or good health (51.9%). About 16.5% described their health as average, while 1.5% rated their health as bad or very bad.

### Translation of the City BiTS-LT

In adherence to ethical research principles, it’s vital for researchers to accurately describe the translation process, addressing challenges encountered and their solutions. Ensuring face validity is crucial to confirm the instrument measures its intended constructs. The City BiTS offers an advantage with its concise item format, typically starting with verb or noun forms, enhancing respondent comprehension. However, some items presented challenges in comprehensive Lithuanian translation due to complex syntactic structures. For example, phrases like ‘Flashbacks to the birth and/or reliving the experience’ and ‘Trying to avoid thinking’ necessitated special considerations. Translation challenges also arose from language-specific idioms that couldn’t be translated directly. For instance, ‘on edge’ was rendered as ‘prie ribos’ in Lithuanian, signifying ‘at the border.'

A third translation challenge involved internationally recognized terms in the City BiTS (e.g., distress, self-destruction, socialization, concentrating), widely understood but requiring Lithuanian alternatives per language guidelines. Balancing native language preservation with instrument applicability, terms like ‘socialization’ became ‘making social activities,’ and ‘distress’ became ‘overstrain,’ with their original synonyms provided. This approach aided comprehension, particularly for individuals with limited literacy.

The choice of precise verbs in translating statements was a key concern. Given the emotional and subjective nature of psychological trauma, selecting verbs and adjectives was vital for question accuracy. Collaborative discussions between Lithuanian researchers and the author of the City BiTS enabled a thorough exploration of meanings, ensuring precise interpretations and avoiding semantic errors. For example, in our case, achieving exact translations was crucial for phrases like ‘feeling negative,’ ‘feeling detached,’ ‘feeling tense,’ and ‘getting upset.’ Equivalence checking revealed disparities in the initial translations. For instance, ‘Did you believe you or your baby would be seriously injured’ was initially translated as ‘Did you worry that you...,’ but concerns about the emotional and anxiety-related aspect of ‘worry’ led to its replacement with ‘think’ for semantic alignment. The focus throughout the translation process was on achieving the best possible semantic match.

### Construct Validity of the City BiTS-LT

After conducting the field study, the instrument’s validity and reliability were assessed. Three CFA models were tested (see Figure 1): a four-factor solution based on DSM-5 dimensions, a two-factor model with birth-related and general symptoms, and a bifactor model with a global factor and two specific factors. For CFA models, good fit criteria include RMSEA < 0.06, CFI and TLI > 0.95, and SRMR < 0.08 (Hu & Bentler, 1999; Kline, 2018; Raykov & Marcoulides, 2012).

The four-factor solution (Model 1) showed poor fit (χ^2^ (164) = 1277.783, *p* < .001; RMSEA = 0.095; CFI = 0.915; TLI = 0.902; SRMR = 0.088) and was rejected. The two-factor model (Model 2) had a good fit (χ^2^ (169) = 497.281, *p* < .001; RMSEA = 0.051; CFI = 0.975; TLI = 0.972; SRMR = 0.053) with moderately correlated birth-related and general symptoms (r = 0.585). Q10 was allocated to Factor 1 due to its weak loadings on both factors and initial as well as post-extraction communalities below 0.2. The bifactor model (Model 3) also had a good fit (χ^2^ (150) = 359.143, *p* < .001; RMSEA = 0.043; CFI = 0.984; TLI = 0.980; SRMR = 0.033) and outperformed the two-factor model (Chi-square difference - 138.138; df difference – 19, *p* < .001). For more detailed information on two-factor and bi-factorial structure see Table 1.

**Table 1.** Factor Loadings for Two-Factor Model and Bifactor Model of the City Birth Trauma Scale (City BiTS)

Items	Model 2		Model 3		
	BRS	GS	GF	BRS	GS
Re-experiencing symptoms					
Q3. Recurrent unwanted memories of the birth (or parts of the birth) that you can’t control	0.788		0.471	0.661	
Q4. Bad dreams or nightmares about the birth (or related to the birth)	0.759		0.636	0.396	
Q5. Flashbacks to the birth and/or reliving the experience	0.661		0.495	0.420	
Q6. Getting upset when reminded of the birth	0.927		0.566	0.743	
Q7. Feeling tense or anxious when reminded of the birth	0.916		0.552	0.743	
Avoidance					
Q8. Trying to avoid thinking about the birth	0.894		0.579	0.685	
Q9. Trying to avoid things that remind me of the birth (e.g., people. Places, TV programmes)	0.842		0.583	0.607	
Negative mood and cognitions					
Q10. Not able to remember details of the birth	0.495		0.495	0.111	
Q11. Blaming myself or others for what happened during the birth	0.765		0.519	0.563	
Q12. Feeling strong negative emotions about the birth (e.g., fear, anger, shame)	0.862		0.577	0.642	
Q13. Feeling negative about myself or thinking something awful will happen		0.718	0.750		0.078
Q14. Lost interest in activities that were important to me		0.767	0.670		0.371
Q15. Feeling detached from other people		0.802	0.693		0.404
Q16. Not able to feel positive emotions (e.g., happy, excited)		0.811	0.650		0.517
Hyperarousal					
Q17. Feeling irritable or aggressive		0.772	0.587		0.554
Q18. Feeling self-destructive or acting recklessly		0.746	0.604		0.461
Q19. Feeling tense and on edge		0.809	0.623		0.572
Q20. Feeling jumpy or easily startled		0.660	0.601		0.264
Q21. Problems concentrating		0.685	0.612		0.299
Q22. Not sleeping well because of things that are not due to the baby’s sleep pattern		0.680	0.694		0.100
Proportion of variance contributed to each set of items by the corresponding latent factor.	*0.64*	*0.56*	*0.63*	*0.65*	*0.58*

Model fit for Model 2 (χ^2^ (169) = 497.281, *p* < .001; RMSEA = 0.051; CFI = 0.975; TLI = 0.972; SRMR = 0.053. Model fit for Model 3 (χ^2^ (150) = 359.143, *p* < .001; RMSEA = 0.043; CFI = 0.984; TLI = 0.980; SRMR = 0.033.

Item-factor analysis showed that Q10 had low factor loading on the specific birth-related symptoms factor, and Q13 and Q22 had low loadings on the general symptoms factor. However, all three items had high loadings on the general factor. Out of 20 items, 13 had higher loadings on the general factor, while 7 had higher loadings on specific factors. The general factor explained nearly two-thirds (63%) of the total common variance, with a greater proportion in items related to birth-related symptoms (52%) than general symptoms (27%).

### Reliability of the City BiTS

Deng and Chan (2017) and Hayes and Countts (2020) argue that McDonald’s omega is a more suitable measure of internal consistency than Cronbach’s alpha. The analysis of the City BiTS-LT and its subscales revealed high reliability with Cronbach’s alpha (α) and McDonald’s omega (ω) values of 0.906 and 0.900 for the entire scale. Inter-item correlations ranged from 0.168 to 0.779. Cronbach’s alpha would only improve if Q10 (‘Not able to remember details of the birth’) were removed.

The birth-related symptom subscale also demonstrated high reliability (Cronbach’s α = 0.885, McDonald’s ω = 0.892), with inter-item correlations ranging from 0.214 to 0.779. Removing Q10 would slightly enhance the subscale’s reliability to 0.895. The internal consistency of the general symptoms subscale was also high (Cronbach’s α = 0.875, McDonald’s ω = 0.881), with inter-item correlations ranging from 0.243 to 0.614. The analysis indicated that removing any questions would not significantly affect the scale’s internal consistency.

### Discriminant Validity of the City BiTS

Discriminant validity of the City BiTS-LT and its subscales was assessed through known-group differences. The results, as presented in Table 2, revealed several significant differences among subgroups. Women who experienced pre-term births exhibited significantly higher levels of birth-related symptoms and obtained higher total scores on the City BiTS-LT compared to women who had term births. Primiparous women reported higher levels of birth-related symptoms and had higher total scores on the City BiTS-LT in comparison to multiparous women. Women who underwent an emergency caesarean section displayed significantly higher birth-related symptoms than those who had spontaneous vaginal deliveries or planned caesarean sections. Additionally, women who underwent instrumental vaginal deliveries exhibited significantly higher birth-related symptoms than those who had spontaneous vaginal deliveries. Furthermore, women who experienced an emergency caesarean section achieved higher total scores on the City BiTS-LT than women who had spontaneous vaginal deliveries.

**Table 2.** Differences in the City Trauma Birth Scale and Subscales Between Known Groups

		Birth-related symptoms	General symptoms	Total score
Groups		*M (SD)*	*M (SD)*	*M (SD)*
Gestational age	Pre-term delivery	7.38 (6.53)	7.40 (6.10)	14.78 (10.50)
	Term delivery	4.03 (5.13)	6.31 (6.20)	10.35 (9.90)
Mann-Whitney, *p*		U = 18661.000, *p* < .001	U = 24670.000, *p* = .055	U = 20182.000, *p* < .001
Parity	Primiparous	4.71 (5.65)	6.66 (6.20)	11.37 (10.39)
	Multiparous	3.81 (4.80)	5.96 (6.09)	9.77 (9.26)
Mann-Whitney, *p*		U = 59678.500, *p* = .014	U = 61203.000, *p* = .057	U = 60015.000, *p* = .021
Type of delivery	Spontaneous vaginal	3.77 (4.94)	6.49 (6.41)	10.27 (9.88)
	Instrumental vaginal	7.93 (6.46)	9.20 (6.60)	17.13 (11.92)
	Emergency c.s.	7.02 (6.07)	7.00 (6.18)	14.02 (10.64)
	Planned c.s.	3.67 (4.77)	6.91 (5.85)	10.58 (8.61)
Kruskall-Wallis, *p*		χ^2^(3) = 32.718, *p* < .001^a^	χ^2^(3) = 3.825, *p* = .281	χ^2^(3) = 14.223, *p* = .003^b^
Traumatic birth (yes/no)	Non-traumatic	3.63 (4.65)	6.17 (6.09)	9.80 (9.18)
	Traumatic	10.10 (6.26)	10.76 (6.81)	20.86 (11.57)
Mann-Whitney, *p*		U = 3847.000, *p* < .001	U = 6420.500, *p* < .001	U = 4750.000, *p* < .001
Newborn’s health	Healthy	4.24 (5.15)	6.35 (6.17)	10.60 (9.88)
	Not healthy	6.87 (6.74)	11.43 (6.96)	18.30 (11.09)
Mann-Whitney, *p*		U = 5636.500, *p* = .054	U = 3975.000, *p* < .001	U = 4199.500, *p* < .001

^a^Emergency c.s > spontaneous vaginal; emergency c.s. > planned c.s.; instrumental vaginal > spontaneous vaginal.

^b^Emergency c.s.>spontaneous vaginal.

Women who perceived their childbirth as traumatic reported significantly higher birth-related symptoms, general trauma symptoms, and total scores on the City BiTS-LT than women who did not consider their childbirth as traumatic. Finally, mothers who reported that their newborns had significant health issues obtained higher scores in general PTSD symptoms and total scores on the scale compared to women whose newborns were healthy

### PTSD Criteria

Table 3 outlines the sample’s adherence to PTSD criteria as per DSM-5. Notably, about 19% of women believed they or their baby might be seriously injured, with 21.8% fearing potential mortality during childbirth. This equated to 28.6% fulfilling PTSD stressor criterion A. A significant majority of women met the criteria for Re-experiencing symptoms (68.53%), while approximately a quarter exhibited Avoidance symptoms (24.83%). Additionally, less than two-thirds experienced Negative cognitions and mood symptoms (61.49%), while Hyperarousal symptoms were present in 58.30% of women. Over half of the women (54.85%) met the criterion for symptom duration exceeding one month, and 47.94% met the distress and impairment criterion. Following the application of exclusion criteria (1.73%) to eliminate symptoms possibly related to medication, substance use, or other illnesses, all DSM Criteria were met by 7.97% of the sample.

**Table 3.** PTSD and DSM-5 Criteria

		‘yes’ answer *N (%)*
Criterion A	Stressor criterion	
	Q1. Did you believe you or your baby would be seriously injured?	143 (19.0)
	Q2. Did you believe you or your baby would die?	164 (21.8)
	Q1 or Q2	215 (28.6)
Criterion B	Re-experiencing symptoms (1 needed) from Q3 to Q7	516 (68.53)
Criterion C	Avoidance symptoms (1 needed) from Q8 to Q9	187 (24.83)
Criterion D	Negative cognitions and mood (2 needed) from Q10 to Q16	463 (61.49)
Criterion E	Hyperarousal (2 needed) from Q17 to Q22	439 (58.30)
Criterion F	Duration (symptoms last for more than 1 month) on Q26	413 (54,85)
Criterion G	Distress and impairment on Q27 to Q28	361 (47.94)
Criterion H	Exclusion criteria on Q29	13 (1.73)
	DSM-5 PTSD	63 (8.37)
	DSM-5 PTSD removing women who meet possible exclusion criteria	60 (7.97)

Furthermore, a majority of women reported experiencing PTSD symptoms within the first six months following childbirth (71.6%). Only 4.2% reported symptoms more than six months post-childbirth, and 8.2% experienced PTSD symptoms before childbirth. While dissociative symptoms are not indicative of PTSD, they enable the diagnosis of a dissociative subtype of PTSD and were reported by 39.18% of the women in the sample.

## Discussion

In this validation study, our aim was to develop and validate a measure of birth-related trauma for use with Lithuanian women following childbirth. The scale underwent a rigorous translation and cultural adaptation process, including addressing phraseology, idioms, and terms/words of international origin, as well as selecting precise terms or verbs to convey the intended emotions. This approach shares similarities with the adaptation of the scale by Brazilian investigators (Donadon et al., 2020), who emphasized semantic equivalence in terms and their meanings, facilitated by researchers’ in-depth language and cultural knowledge, along with active involvement from the original instrument’s author.

Confirmatory factor analysis verified the validity of a two-factor and bifactor model, with the bifactor model offering the best fit. This bifactorial model had initially been tested in the Croatian sample, demonstrating superior fit compared to a two-factor model. In both the Croatian and our current study, the proposed four-factor PTSD structure outlined in DSM-5 failed to achieve satisfactory fit (Nakić Radoš et al., 2019).

Our findings also indicated that the City BiTS-LT exhibits high internal reliability for the total scale and its subscales. Notably, the item ‘Not able to remember details of the birth' displayed weak loadings on both factors, aligning with results from other validation studies (Ayers et al., 2018; Nakić Radoš et al., 2019). This outcome could be attributed to the significance of childbirth as a life-changing event, making women (and their partners) more likely to remember details compared to other traumatic stressors. Alternatively, the impact of pain, analgesia, or anesthesia on memory may play a role. Several factors, such as complex Caesarian sections, fatigue, sleep deprivation, stress, medical procedures, high emotional intensity, and others, could affect memory. Future research should delve into these factors to gain a more comprehensive understanding of their influence on birth-related memory.

Our study unveiled that birth-related symptoms displayed greater sensitivity to gestational age, parity, birth type, and traumatic birth experiences. We observed significantly higher birth-related symptoms in women who had preterm births, were primiparous, underwent emergency caesarean sections, had instrumental vaginal deliveries, or described their birth as traumatic. In contrast, general trauma symptoms exhibited higher sensitivity when considering newborn health and the subjective perception of birth as traumatic. These findings align with the Croatian validation study, which also identified the birth-related symptoms subscale as more sensitive to birth type and parity (Nakić Radoš et al., 2019). Overall, our results confirm that women perceiving their birth as traumatic face an elevated risk of developing PTSD (Bayri Bingol et al., 2021). However, it’s worth noting that the association between obstetric variables and City BiTS subscales varies across different studies. For instance, Weigl et al. (2021) found that emergency Caesarean sections were related to higher scores on birth-related and general trauma symptoms, as well as the total scale score.

Descriptive analysis of PTSD symptoms in Lithuanian women corresponds to a UK study assessing the original version of City BiTS (Ayers et al., 2018). In both cases, around 7.97% of the Lithuanian sample and 7.1% of the UK sample met the diagnostic criteria for PTSD, fulfilling all DSM criteria. However, a German study reported a much lower percentage, with only 2.8% of women meeting the criteria for a PTSD diagnosis (Weigl et al., 2021). These differences may be attributed to varying sampling methods and potentially distinct clinical characteristics among participants. For instance, the Lithuanian sample had a notably higher prevalence of preterm births compared to the German sample. Our study’s self-selected participant approach indicates that they may not represent the broader Lithuanian population accurately. Therefore, further research based on population-wide sampling is essential to determine the prevalence of birth-related PTSD among Lithuanian women. Comprehensive investigations are required before drawing definitive conclusions (Osório et al., 2021).

This study has several limitations. A substantial portion of participants were recruited through online and social media platforms, making it challenging to determine the response rate of the potential sample. The self-selection process and limited personal data collected further hinder our ability to assess the representativeness of the sample. Additionally, similar to findings in other studies (Ayers et al., 2009; Nakić Radoš et al., 2019), online-recruited samples may exhibit a higher risk profile, potentially inflating estimates of PTSD prevalence. While this study followed proper psychometric procedures, it did not include a test-retest measurement, which limits our ability to draw conclusions about the stability of the City BiTS-LT over time.

In summary, this study has confirmed that the Lithuanian version of the City Birth Trauma scale is a valid and reliable instrument for the clinical and scientific evaluation of trauma symptoms in postpartum women. It offers versatility, allowing it to be used either as a comprehensive total scale or as informative subscale indicators, with the bifactorial structure adding to its reliability by providing two subscales in addition to an overall score. This standardized and high-quality assessment tool also facilitates international comparisons of research findings and the exploration of cultural differences in women’s perceptions of negative birth experiences.

Our findings underscore the need for clinical awareness regarding birth trauma and its intricate connection to a woman’s perinatal mental health. Regrettably, birth trauma often goes unnoticed. The absence of standardized procedures for systematically assessing women’s psychological well-being during the crucial postnatal period means that concerns are typically addressed only when women voluntarily report symptoms or complaints. Tragically, the absence of a platform for women to express their traumatic experiences frequently results in inadequate support (Elmir et al., 2010). To enhance perinatal care, it is imperative to systematically assess women’s birth experiences using valid and reliable measures. Future validation efforts for this instrument should encompass diverse sampling methodologies and involve the comparison of symptom experiences among more homogeneous groups of women based on childbirth type, levels of healthcare services, and women’s subjective and objective health statuses.

## Supplemental Material

Supplemental Material - Translation and Validation of the City Birth Trauma Scale With Lithuanian Postpartum Women: Findings and Initial Results Supplemental Material for Translation and Validation of the City Birth Trauma Scale With Lithuanian Postpartum Women: Findings and Initial Results by Olga Riklikienė, Gabija Jarašiūnaitė-Fedosejeva, Ernesta Sakalauskienė, Žydrūnė Luneckaitė, and Susan Ayers in Evaluation & the Health Professions
