# Supplementary material for: Translation and Validation of the City Birth Trauma Scale With Lithuanian Postpartum Women: Findings and Initial Results
Source: Eval Health Prof. 2024 Mar 12;48(2):213–21. doi: 10.1177/01632787241239339 (PMC11979301; doi:10.1177/01632787241239339)
Supplement: Supplemental Material - Translation and Validation of the City Birth Trauma Scale With Lithuanian Postpartum Women: Findings and Initial Results [file sj-pdf-1-ehp-10.1177_01632787241239339.pdf]

Supplementary Material.

Figure 1

Four-factor, ***two-factor and bifactor structure of analyzed models***

Model 1 **Model 2** **Model 3**

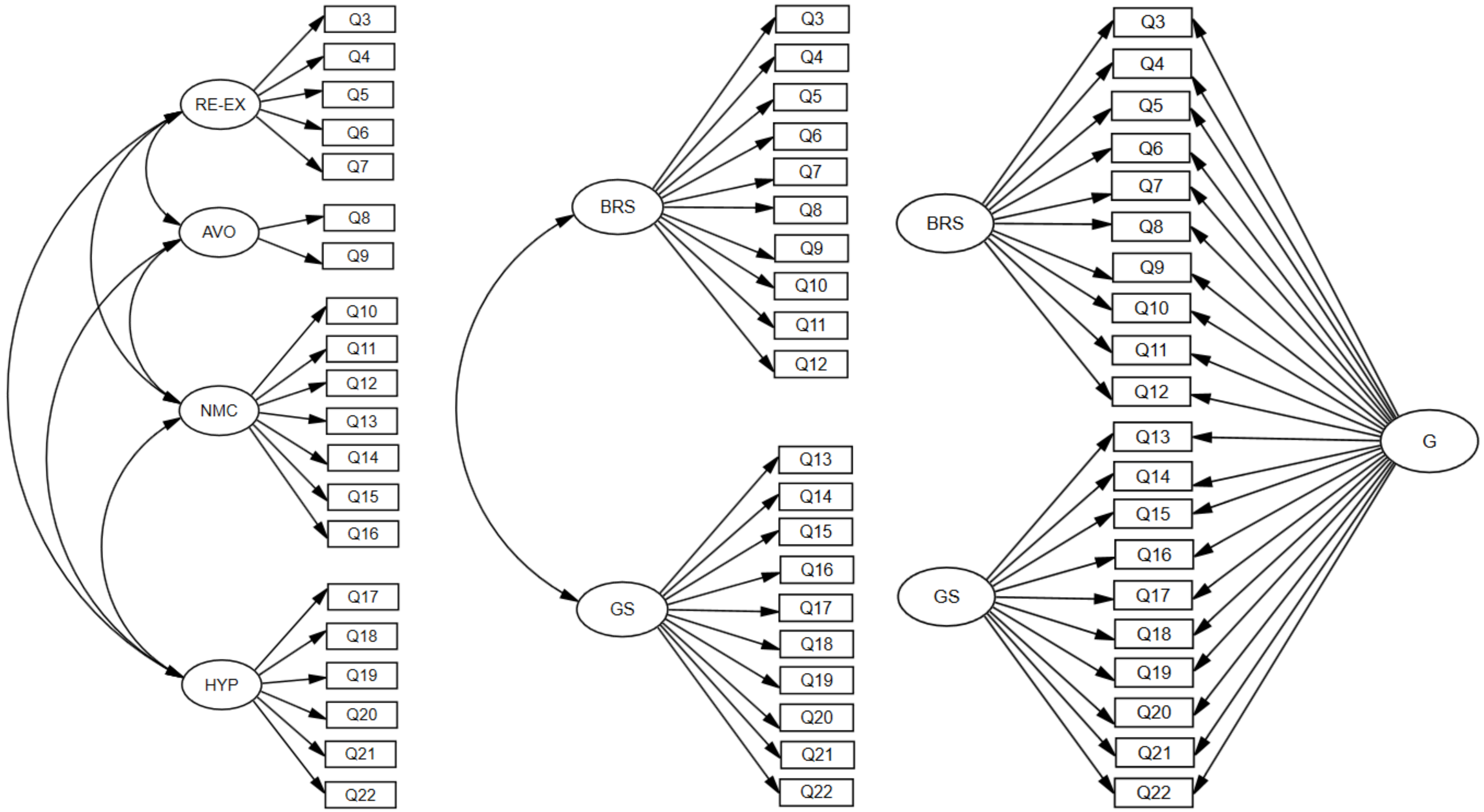

Note. Four-factor correlated model (Model 1) with four clusters from DSM-5 including Re-experiencing symptoms or Intrusions (RE-EX), Avoidance (AVO), Negative mood and cognitions (NMC), and Hyperarousal (HYP). Two-factor correlated model (Model 2) with specific factors of Birth-related symptoms (BRS) and General symptoms (GS). Bifactor model (Model 3) with the global factor (G) and specific factors of Birth-related symptoms (BRS) and General symptoms (GS).

Table 4

Exploratory Factor Analysis Outcomes

Items	***Factor1***	***Factor2***
Birth-related symptoms		
Q6. Getting upset when reminded of the birth	*0.870*	
Q7. Feeling tense or anxious when reminded of the birth	*0.856*	
Q8. Trying to avoid thinking about the birth	*0.806*	
Q12. Feeling strong negative emotions about the birth (e.g., fear, anger, shame)	*0.790*	
Q3. Recurrent unwanted memories of the birth (or parts of the birth) that you can't control	*0.720*	
Q9. Trying to avoid things that remind me of the birth (e.g., people, places, TV programs)	*0.663*	

Q11. Blaming myself or others for what happened during the birth	*0.630*	
Q5. Flashbacks to the birth and/or reliving the experience	*0.475*	
Q4. Bad dreams or nightmares about the birth (or related to the birth)	*0.448*	
Q10. Not able to remember details of the birth	*0.213*	*0.244*
General symptoms		
Q19. Feeling tense and on edge		*0.790*
Q16. Not able to feel positive emotions (e.g., happy, excited)		*0.753*
Q15. Feeling detached from other people		*0.737*
Q17. Feeling irritable or aggressive		*0.731*
Q14. Lost interest in activities that were important to me		*0.684*
Q21. Problems concentrating		*0.597*
Q20. Feeling jumpy or easily startled		*0.531*
Q13. Feeling negative about myself or thinking something awful will happen		*0.506*
Q18. Feeling self-destructive or acting recklessly		*0.505*
Q22. Not sleeping well because of things that are not due to the baby's sleep pattern		*0.473*
% of variance explained	*37.13*	*13.04*
